# Use of Short Messaging Service to Improve Follow-Up for Abnormal Pap Test Results in Minority and Medically Underserved Women in North Carolina: Questionnaire on Attitudes and Acceptability

**DOI:** 10.2196/12675

**Published:** 2019-08-06

**Authors:** LaHoma Smith Romocki, Andrea Des Marais, Leslie Cofie, Chelsea Anderson, Theresa Curington, Jennifer Susan Smith

**Affiliations:** 1 Department of Public Health Education North Carolina Central University Durham, NC United States; 2 Department of Epidemiology Gillings School of Global Public Health University of North Carolina at Chapel Hill Chapel Hill, NC United States; 3 Department of Health Education and Promotion East Carolina University Greenville, NC United States; 4 School of Medicine Duke University Durham, NC United States

**Keywords:** cervical cancer, Pap tests, abnormal results, text messaging, appointment reminders

## Abstract

**Background:**

An estimated one in eight cervical cancer cases are due to a lack of follow-up care for abnormal Pap test results. Low rates of completion of follow-up care particularly affect low-income minority women. The burden of cervical cancer could be reduced through interventions that improve timely colposcopy follow-up and treatment of abnormal screening results. Mobile communications via text messaging present a low-cost opportunity to increase rates of clinic return among women referred to follow-up after obtaining abnormal screening results.

**Objective:**

Our aims were to determine the acceptability and feasibility of using text messaging to increase completion of follow-up care following abnormal cervical cancer screening (Pap test) results and to examine factors that may affect the acceptability and use of text messaging to increase communications between health care providers (HCP) and low-income minority women.

**Methods:**

The study participants were 15 low-income women who had undergone a Pap test within the preceding 12 months. Semistructured interviews, including open- and closed-ended questions from a validated questionnaire, were conducted by phone or in person. Responses to closed-ended survey items were tabulated, and descriptive statistics were generated using Microsoft Excel. Responses to the open-ended questions were coded and analyzed using NVivo 11 qualitative analysis software.

**Results:**

Nearly all participants (14/15, 93%) were comfortable receiving a text message from an HCP stating that their Pap test results were available (<40 years: 100%; ≥40 years: 86%). Over half (8/15; 53%) of the participants were comfortable receiving a text message stating that their Pap test results were abnormal, although many preferred to receive such information via a phone call (6/15; 40%). Most participants (9/15; 60%) believed that receiving a text reminder would make them more likely to attend their appointment. The preferred method for receiving a reminder appeared to vary by age, with older women preferring telephone reminders over text messaging reminders. Analysis of open-ended questions suggested that text messaging appeals to some women due to its wide use and convenience for communicating with HCPs. However, women cited concerns about the confidentiality of messages and barriers to understanding the messages, including the physical capacity to read and accurately interpret the content of the messaging.

**Conclusions:**

Most participants indicated a willingness to receive text messages from their HCPs about cervical cancer screening results and believed that text messages were the best way to remind them of appointments for follow-up care. Potential concerns could be addressed by excluding explicit references to the nature of the appointment in the text message in order to avoid disclosure of sensitive health information to unauthorized individuals. Although text messaging seems promising to improve adherence to timely follow-up, personal preferences should be considered by allowing patients to opt-out of text communications.

## Introduction

Cervical cancer mortality rates are significantly higher in African American/black women than any other racial or ethnic group in the United States [1,2]. This health disparity persists, especially in the South [3], despite evidence that black non-Hispanic woman participate in primary cervical cancer screening activities at approximately the same rates as white non-Hispanic women [4]. Regular cotesting with Pap tests (cytology) and oncogenic types of human papillomavirus (HPV), the virus that causes cervical cancer, is the primary screening method recommended to detect the presence of abnormal precancerous lesions [5].

In the United States, recommended follow-up for abnormal Pap results is completion of colposcopy with biopsy to confirm the presence of a high-grade lesion requiring treatment (cervical intraepithelial neoplasia, grade 2 or more severe, CIN2+), followed by treatment of CIN2+ through outpatient procedures such as loop electrosurgical excision procedure or cold cone biopsy [6]. Progression to invasive cervical cancer (ICC) can be prevented with the early detection of high-grade lesions and adherence to follow-up recommendations for further diagnostics and treatment [6]. Thus, the burden of cervical cancer could be significantly reduced through interventions that promote timely follow-up and treatment of women with abnormal Pap results [7].

Standard clinical protocols require that patients are notified of abnormal Pap results and referred to appropriate follow-up colposcopy and treatment procedures. However, multiple and complex factors may contribute to patient nonadherence to recommended follow-up once patients are informed of their abnormal screening results. These factors include patients’ lack of the following factors: knowledge about HPV infection and cervical cancer [8], understanding about the seriousness of an abnormal Pap diagnosis [9], trust in health care providers (HCPs), health insurance, and family and social support [10,7]. Another important challenge is the inability to inform the patient of an abnormal result in a timely fashion due to a disconnected or changed phone number or an incorrect address to send or forward screening results [11]. Any one of these factors may lead a woman to miss or delay her follow-up care, increasing her chances of a poor clinical outcome.

Racial and ethnic differences have been observed in follow-up rates after an abnormal Pap test [12-15], in which African American and Hispanic women were less likely to schedule follow-up visits and receive follow-up care. In contrast, other studies have shown differences in follow-up rates between races to be negligible or even nonexistent [16]. It is imperative that women at the greatest risk of cervical cancer receive follow-up care after an abnormal Pap test or high-risk HPV test result to reduce cancer disparities. This can be achieved by increasing follow-up visits in this higher risk population [5].

The rapid expansion of mobile communications may present a low-cost opportunity to improve follow-up rates. Following an abnormal Pap test result, patients who receive a reminder via text message or short messaging service (SMS) may be more likely to attend a follow-up visit. SMS interventions have been shown to improve the rate of attendance at health care appointments for surveyed populations [17] and may have a similar impact as phone call and postal reminders [18-20]. With respect to cervical cancer, a prior study reported on the use of an SMS intervention to increase knowledge and encourage Pap test screening in Korean American [21] and African American women [22]. However, the feasibility of an SMS intervention to improve follow-up rates for in-clinic colposcopy attendance after an abnormal Pap test result has not been previously explored. For such an intervention to be successful among low-income and minority women, research is needed to understand appropriate SMS-based content, messaging, and context [23] and to determine the likelihood that such an approach would be acceptable to this population of patients.

Therefore, the objective of this study was to explore factors that may play a role in the use of text messaging as a mode to increase communications between HCPs among low-income and minority women who have undergone Pap tests as well as the need to return to the clinic to prevent potential advanced cancers.

## Methods

### Study Design

Participants were recruited from two clinics serving low-income and minority populations—a free community-based clinic and a university-based clinic—both of which are located in Durham, North Carolina. Women were eligible to participate in the study if they were between the ages of 21 and 60 years and had undergone a Pap test in the preceding 12 months. Participants were recruited by collaborating HCPs who provided eligible patients written information about the study and flyers with contact information at the time of their clinic appointment. Interested women signed forms giving permission for the research team to contact them and were contacted by phone for study enrollment. Due to challenges in successfully enrolling women through this process (eg, wrong telephone numbers, failure of participants to reply to messages, and difficulty scheduling appointments), the recruitment strategy was shifted to screen, enroll, and interview patients before or immediately after examination by the HCP, unless patients preferred to be contacted at a later time. The clinic provided a room where interviews could be conducted confidentially. This approach greatly reduced the loss between referral from the HCP and successful study completion within the allotted study period.

### Interviews

After the participants completed an informed consent, semistructured interviews were conducted over the phone or in person, depending on the preference of the participant. The 58-item interview guide included items from a review of the literature of SMS interventions and a validated questionnaire exploring attitudes and knowledge about HPV and cervical cancer. The combination of open- and closed-ended items included topics on overall attitudes of patients about text messaging practices, relationship with HCPs, preferences about clinic and appointment reminders, and knowledge about cervical cancer and HPV. Interviews were audio recorded and transcribed.

### Data Analyses

Responses to closed-ended survey items were tabulated, and descriptive statistics were generated using Excel to report the demographic data, texting practices, and preferences variables. Responses to the open-ended questions were examined by two experienced qualitative researchers during four stages of analysis. As part of the first stage, transcripts were entered into NVivo (version 11; NVivo qualitative data analysis software; QSR International Pty Ltd),

a qualitative software analysis package, and organized to allow each reader to efficiently access the same data while allowing for separate sets of analyses. Second, each reader independently read the original transcripts line by line to identify general themes or codes that emerged from the data using the memo functions and other tools of the software. Third, the readers compared their interpretations and refined themes that could be confirmed and systematically evidenced by the data. Fourth, the coders agreed on a final set of themes that appeared to be the most relevant to the study participants based on the frequency of appearance and richness of the evidence.

## Results

### Participants

The median age of the 15 participants was 34 years (range: 21-58 years). Most women were of African American race (n=13, 87%), had at least some college education (n=10, 57%), and were unemployed (n=9, 60%). In addition, a majority had health insurance (n=10, 67%), most through Medicaid, and nearly all owned a cell phone (n=14, 93%).

### Cell Phone Use

Of the 14 women who own a cell phone, 13 (93%) used text messaging, with some differences seen in the frequency of use according to age. All 10 women who had at least some college education (100%) and most women with a high school degree or less education (4/5, 75%) used their cell phone to send text messages (Table 1).

**Table 1:** Sociodemographic characteristics and cell phone utilization.

Characteristic		Overall (N=15)	Age <40 years (N=8)	Age ≥40 years (N=7)	Fisher exact *P* value
Age (years), median (range)		34 (21-58)	24 (21-34)	50 (46-58)	N/A^a^
**Race, n (%)**					>.99
	African American	13 (87)	7 (88)	6 (86)	
	White	2 (13)	1 (13)	1 (14)	
**Education, n (%)**					>.99
	Less than or high school degree	5 (33)	3 (38)	2 (29)	
	Some college/college degree	10 (57)	5 (63)	5 (71)	
Employed, n (%)		6 (40)	4 (50)	2 (29)	.61
Has health insurance, n (%)		10 (67)	5 (63)	5 (71)	>.99
Owns a cell phone, n (%)		14 (93)	7 (88)	7 (100)	>.99
**Cell phone use per day, n (%)^b^**					.001
	≤10	6 (43)	0	6 (86)	
	>10	8 (57)	7 (100)	1 (14)	
**Text messages received per day, n (%)^b^**					.20
	<25	11 (79)	4 (57)	7 (100)	
	≥25	3 (21)	3 (43)	0	
**Text messages sent per day, n (%)^c^**					.46
	<25	11 (85)	5 (71)	6 (100)	
	≥25	2 (15)	2 (29)	0 (0)	
**Reported phone uses, n (%)^b,d^**					
	Send text messages	13 (93)	7 (100)	6 (86)	>.99
	Find directions	10 (71)	6 (86)	4 (57)	.56
	Take photos	13 (93)	7 (100)	6 (86)	>.99
	Check Facebook or Twitter	10 (71)	7 (100)	3 (43)	.07

^a^N/A: not applicable.

^b^N=14 women who owned a cell phone (n=7 for women aged <40 years and n=7 for women aged ≥40 years).

^c^N=13, missing one response (n=7 for women aged <40 years and n=6 for women aged ≥40 years).

^d^More than one choice could be selected.

### Communication Preferences

Most women (n=14, 93%) indicated that they were comfortable receiving text messages from their HCPs following primary screening visits. Nearly all women in the study (n=14, 93%) were comfortable receiving a text message from an HCP, stating that their Pap test results are available. However, 75% (6/8) of women aged <40 years and only 29% (2/7) of women aged ≥40 years were comfortable receiving a text message stating that their Pap test results were abnormal (Table 2).

When offered a list of preferences for receiving information about their abnormal Pap test results (including via phone, text, letter, or some other way), 6 (43%) of the 14 participants who owned cell phones preferred to receive this information by phone, 2 (14%) preferred text, and 6 (43%) stated no preference.

Nearly half (7/15, 47%) of all participating women were comfortable receiving a reminder via phone to come back to the clinic for follow-up care after an abnormal Pap test, and 33% were comfortable receiving a reminder by text message. Most participants (13/15, 87%) indicated that if they received a text message from the clinic, they would be willing to respond to indicate that they had received the message (Table 2).

**Table 2:** Women’s communication preferences for test results overall and stratified by age. No significant relationships were found (using the Fisher exact test).

Preference		Overall (N=15), n (%)	Age <40 years (n=8), n (%)	Age ≥40 years (n=7), n (%)	Fisher exact *P* value
**Comfortable receiving information that lab test results are available through:**				
	Phone message	15 (100)	8 (100)	7 (100)	>.99
	Text message	13 (87)	8 (100)	5 (83)	.20
**Comfortable receiving a text message from a health care provider stating that Pap test results are:**				
	Available	14 (93)	8 (100)	6 (86)	.47
	Abnormal	8 (53)	6 (75)	2 (29)	.13
Willing to respond to indicate that you had received a text message from the clinic		13 (87)	8 (100)	5 (71)	.20

### Attitudes About Communication Preferences

Analysis of open-ended questions provided useful details on the benefits and barriers to using text messaging for communicating with HCPs, receiving test results, and receiving follow-up visit reminders (Table 3). Cited advantages of text messaging included ease of use and convenience, especially to receive notifications. For some women, texting was no different than the current common practice of providing appointment reminders by phone. However, several participants (particularly older participants) reported barriers to text messaging, including inconvenience (it is easier to call), lack of texting proficiency (not comfortable with texting, texting too slowly, or technologically challenged), difficulty in texting (diminished physical ability), and a lack of confidence in their ability to correctly understand and interpret the content of the text message (often due to low literacy). Some participants felt that the text message would be useless because they generally ignore unsolicited text messages from a source unknown to them.

Most participants also reported concerns over privacy and confidentiality of health information communicated by text, such as receiving a notification for a positive Pap test or for a follow-up visit after a positive Pap test. Most participants were concerned that family members, friends, coworkers, or strangers could gain access to personal health information if the phone was lost or left unattended or was used by others (eg, some participants share their phone with family members). Some participants were also concerned about the potential of hackers accessing their private health information. When probed to explore what might allay their privacy concerns, participants seemed open to discussing strategies such as encryption or passwords, although some still expressed skepticism about the ability of the clinic to safeguard the information.

**Table 3:** Emergent themes: attitudes about receiving text message reminders to return to the clinic to follow-up on abnormal cervical cancer test results.

Theme		Response
**Benefits**		
	Convenience	“Text just makes it easier to check and see, and then it would be helpful if the text contained a number to call back.” [P^a^14]
	Notification	“It’s impersonal, but just to text me to inform me to come in or your results is back, that’s notification.” [P7]
**Barriers**		
	Capacity to text (knowledge, comfort, and ability)	“It’s a lot of text messages on here, but I don’t know how to pull it up on my phone. I just don’t know how to do it.” [P9] “I’m not a text person myself. To me – texting is for the younger generation. It takes more time to text than to just call.” [P3]
	Physical limitations	“My hand get tired and I have carpal tunnel so I don’t mess with it...And then I have arthritis in my hands too, so can’t do that...” [P3]
	Trust	“I have seen some messages about different things but like I said, if it was unsolicited, I didn’t pay it any attention.” [P10]
	Confidentiality and privacy	“Let’s say for example, I leave my phone sitting right here and I go to the bathroom, then all of a sudden, they decided to text me and the text is gonna show up on my phone.” [P10] “Some people don’t have a lock on their phone like I don’t have a lock on my phone.” [P12] “It’s hackers out there and...they go into you medical history and they can pull it up and see everything right there on the phone.” [P8]

^a^P: participant.

## Discussion

In this study, we determined the feasibility and acceptability of using text messaging to improve abnormal Pap test follow-up in minority and medically underserved women in North Carolina. Overall, most respondents seemed receptive to text messaging reminders from their health care providers. Most participants were open to receiving text message notifications that their tests results were available as well as text message reminders about the need to come back to the clinic for follow-up appointments. More respondents preferred receiving abnormal Pap test results by phone rather than by text, although a large proportion reported no preference.

Although most participants reported finding text messaging easy to use and convenient, some participants reported barriers to using text messages (such as comfort, knowledge, and literacy) and concerns about the confidentiality of their private health information. To address issues of confidentiality, clinics could minimize details in the message about the nature of the test results or appointment. Personal preferences should be considered in the use of this technology by allowing patients to opt-out of text communications. Incorrect contact information is a challenge for health care institutions to ensure adequate follow-up, which might be addressed by verbal communication or text message confirmation of the correct number while the patient is still at the clinic for her screening appointment.

A strength of this study lies in the use of open-ended and closed-ended items, which allowed the researchers to both determine the proportion of particular attitudes and collect more detailed information on the benefits of using text messaging to increase compliance with HCP instructions and guidelines as well as the challenges and barriers that may need to be overcome to maximize the use of this technology in this population. Many cognitive and personal factors are associated with failure to comply with recommended follow-up on abnormal results [9-11], which represent broader contextual issues for consideration. For example, one limitation of this study is that we did not systematically collect data on previous experience with abnormal Pap results to assess how these experiences might affect current attitudes.

This study has other limitations. First, due to the small sample size and the nature of the population enrolled (majority of minority and medically underserved women in North Carolina), participants’ opinions collected in this study may not reflect those of a broader population. Second, although the focus of the study was to determine the acceptability of text messaging among women who had already undergone primary screening, the importance attributed to the use of all mobile phone apps, including social media platforms (ie, Facebook) particularly for younger women who are more accustomed to these technologies, was not examined. Third, further research is needed to study the effects of such apps on secondary and tertiary prevention activities.

Successful follow-up care and treatment of women with abnormal Pap test results are important for preventing ICC. Reminders are useful tools, which have shown to improve regular screening practices and may allow health care providers to improve adherence to follow-up recommendations. Mobile phones are ubiquitous and provide a convenient and low-cost approach to improve adherence rates. Further research in an actual intervention study is needed to assess the feasibility and effectiveness of SMS approaches to improve adherence to follow-up care among high-risk women with abnormal cervical cancer screening results. A trial is in development to evaluate the effect of a text message intervention on increasing follow-up completion.

## Abbreviations

HCP: health care providers
HPV: human papillomavirus
ICC: invasive cervical cancer
SMS: short messaging service
